# Challenges of transferring rural adults with chronic HCV infection for further HCV RNA confirmation and free DAAs treatment: a success story of the interdisciplinary collaboration approach

**DOI:** 10.1186/s12879-020-05435-3

**Published:** 2020-10-07

**Authors:** Wei Li, Te-Sheng Chang, Shu-Zhi Chang, Ching-Hwa Chen, Mei-Yen Chen

**Affiliations:** 1 Formosa Plastics Group Health Care, Yunlin, Taiwan; 2 Department of Internal Medicine, Division of Gastroenterology and Hepatology, Chang Gung Memorial Hospital, Yunlin, Taiwan; 3 Formosa Plastics Group, Advanced Engineer, Safety Health and Environment Center, Taipei, Taiwan; 4 Department of Nursing, Chang Gung University of Science and Technology, Chiayi, Taiwan; 5 Research Fellow, Department of Cardiology, Chang Gung Memorial Hospital, No. 2, Chiapu Rd. West Sec., Putz City, Chiayi County, 613 Taiwan; 6 School of Nursing, Chang Gung University, Taoyuan, Taiwan

**Keywords:** Hepatitis C elimination, Anti-HCV positive, Direct-acting antivirals, Barriers, Facilitators, Rural

## Abstract

**Background:**

Chronic hepatitis C virus (HCV), which is a concern in many countries, is the leading cause of liver cancer around the world. Since Taiwan launched its national health insurance system in 1995, it has managed to extend health coverage to 99% of the Taiwanese population, providing free but limited antiviral treatment each year since 2017. However, many people in rural areas are unaware that they have chronic HCV; nor do they realize that new drugs with high cure rates could drastically reduce their health burden. The aim of this study is to explore the implementation facilitators of and barriers to inviting potentially infected patients in rural areas to be transferred for HCV ribonucleic acid (RNA) confirmation and new drug treatment.

**Methods:**

A descriptive and prospective study design with an interdisciplinary collaboration approach was implemented. After five elements of referral were developed, telephone counseling was conducted between August 2018 and May 2019 in Yunlin, Taiwan. The elements of referral developed by the research team were: (1) forming and coordinating physicians’ schedules, (2) recruiting and training volunteers, (3) training the nursing staff, (4) raising funds or resources, and (5) connecting with village leaders. Thereafter, we collaborated with two district health centers, a private local hospital, and health clinics. Based on the medical records provided by these agencies, community adults that were HCV antibody (anti-HCV) positive were invited to join the program.

**Results:**

Of the 1795 adults who were serum anti-HCV positive, 1149 (64%) accepted transfer to a qualified hospital; of these, 623 (54.2%) had an HCV infection. 552 (88.6%) of those infected started receiving direct-acting antivirals (DAAs) treatment. The top four barriers to accepting transfer were: (1) they perceived themselves to be healthy (*n* = 98, 32.3%); (2) mistrust of treatment/healthcare (*n* = 60, 20.2%); (3) limited transportation to the hospital (*n* = 52, 17.5%); and (4) work conflict (*n* = 30, 10.1%).

**Conclusion:**

An interdisciplinary collaboration approach significantly contributed to the invitation of CHC patients, as well as their acceptance of HCV RNA confirmation and free DAAs treatment. Using anti-HCV data from previous medical records for case-finding and collaborating with a hospital and health clinics proved to be an efficient strategy.

## Background

According to the World Health Organization [1], more than 325 million people worldwide are chronically infected with hepatitis B or C virus (HBV or HCV). In many countries, mass infant vaccination has helped control HBV. However, no vaccine has been found to prevent HCV infection [2]. HCV is a blood-borne virus. For some people, it is a short-term illness; for more than 50% of people infected with HCV, however, it is a long-term, chronic infection [2]. Chronic hepatitis C (CHC) is a concern in many countries, and is the leading cause of liver cirrhosis and hepatocellular carcinoma worldwide [3]. CHC, which is defined as being infected with HCV for more than 6 months, is responsible for 57% of cases of liver cirrhosis, 78% of cases of hepatocellular carcinoma, and an estimated 1.3 million preventable deaths a year worldwide [4]. Across the globe, approximately 71 million people are infected with CHC [5]. Research indicates that once a person is infected with HCV, it becomes chronic in 75–85% of cases— with a high probability of progressing to liver cirrhosis and hepatocellular carcinoma [3]. Since the liver has no nerve endings and can function despite 70% of its mass being damaged, liver diseases are commonly referred to as “silent killers” and are often asymptomatic [4, 6].

The average prevalence rate of HCV infection in Taiwan is 3.87% [6]. The rate is even higher (10%) in the country’s southwestern coastal rural areas, making it significantly higher than those in the United States and European countries [1, 7]. This phenomenon was initiated five decades ago when many rural residents of Taiwan were treated with inadequately disinfected medical equipment while sick. For instance, they were given injections by unqualified physicians, which caused many innocent people to be unknowingly infected by HCV [6, 8]. It is estimated that 400,000–700,000 people in Taiwan have CHC. Patients with CHC have been treated with interferon-based therapies, which are commonly associated with adverse effects and long treatment duration [8]. Fortunately, the emergence of direct-acting antiviral (DAA) agents has proven to be a highly effective cure that also maintains patient safety [2, 6, 9–11]. The Taiwanese government has initiated a national elimination goal of eradicating CHC by 2025, which will be paid for by the Taiwan National Health Insurance (NHI). The free DAAs treatment was launched in 2017, with a maximum of around 11,000 treatments being provided annually, and each treatment costing 10,000 USD [6, 12].

Previous studies have indicated that universal HCV screening for adults, rather than just for birth cohorts and high-risk populations, is a key intervention tool used to inform people of their hepatitis status, and direct and motivate those infected to obtain the necessary medical care [4, 13, 14]. Given the costs, there are usually two steps to screen and confirm for an active HCV infection. The first step is to encourage people to participate in blood screening for anti-HCV; this is because being serum anti-HCV positive indicates either an active or a resolved HCV infection. If this test is positive, a confirmatory test—the HCV ribonucleic acid (RNA) test, also known as the HCV viral load test—is performed in order to identify active CHC infection [1, 15].

Although evidence indicates that DAA treatment for HCV has been successful, involves few side effects, and is free of charge through Taiwan’s NHI [6, 8], many people with HCV do not have sufficient knowledge regarding liver health and treatment. For instance, Lin et al. [4] found that people who underwent blood screening for anti-HCV did not know the results nor their hepatitis status. Indeed, there is a gap that needs to be addressed in order to achieve the national elimination goal of eradicating HCV [4, 14, 16]. In this regard, primary healthcare providers—who work at the frontline of the healthcare system—play an important role in actively promoting the free DAAs policy of the Taiwanese government. They should refer people with chronic HCV infections in rural areas for transfer to a qualified hospital for HCV RNA confirmation and free DAAs treatment. The aim of this study is to explore the implementation facilitators of and barriers to inviting potentially infected chronic HCV patients in rural areas to accept transfers for HCV RNA confirmation, and encouraging those who are HCV RNA positive to avail of free DAAs treatment.

## Methods

### Design, sample, and setting

A descriptive and prospective study design with an interdisciplinary collaboration approach was implemented between August 2018 and May 2019 in coastal Yunlin County. The researchers collaborated with a local hospital, three private outpatient clinics, and two district health centers that have been providing health screenings to community residents around the coastal western areas for over 10 years. All participants over 20 years old who were anti-HCV positive according to the medical records were invited to the collaborating hospital for further HCV RNA confirmation.

### Ethical considerations

This study was approved by an ethical committee (Institutional Review Board No. 201701919B0) and was conducted in accordance with the principles of the Declaration of Helsinki (2008).

### Procedure

The interdisciplinary collaboration approach, which involved five elements of referral and telephone counseling, was established by the research team as follows:

1. Forming and coordinating physicians’ schedules: Five gastroenterology and hepatology experts were invited to join the program and reach a consensus on HCV confirmation and the treatment protocol for people potentially infected with chronic HCV. The research team arranged five physicians’ clinic schedules from Monday through Saturday based on the convenient outpatient time of the people invited for HCV RNA confirmation. Once HCV RNA positivity was confirmed, the patients would be persuaded by trained nursing staff to avail of the free DAAs treatment over a period of 8 ~ 12 weeks, depending on one’s genotype.
2. Training the nursing staff to communicate four points during the telephone interview: Five nurses were trained to perform telephone interviews by emphasizing the following points: (a) treatment is provided for free: a free DAAs treatment saves patients at least 10,000 USD; (b) full understanding of the mechanism of HCV; (c) encouragement to join the national HCV elimination program; and (d) the reduction of transportation or transfer barriers. In addition, the nursing staff were trained to discuss the following: (a) who and where I am, (b) how I obtained your data, (c) why I called you, and (d) one call every other day during the first two weeks after CHC patients start treatment in order to make sure they take the oral drugs;
3. Recruiting and training volunteers: Eight senior nursing students were trained to guide a resident when he or she was referred to a hospital for HCV confirmation and/or treatment;
4. Raising funds or resources: Resources were collected, for example, for some poor families in order to resolve their transportation issues; and.
5. Connecting with village leaders: The research team met with village leaders to ask them to publicize this project in all villages and assure community residents that a telephone call about the project would be from the research team and not a phone scam.

### Data analysis

**Fig. 1:**
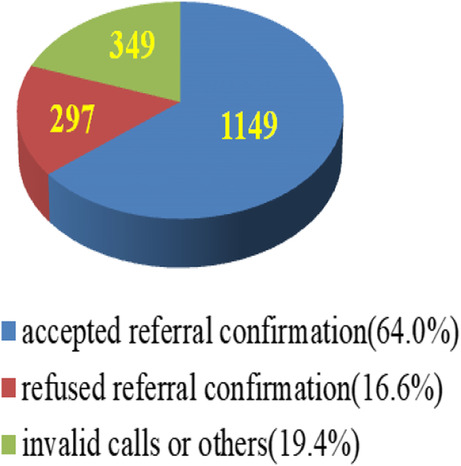
Anti-HCV positive (*n* = 1795)

Microsoft Office Excel (2007) was used to record the demographic information of anti-HCV positive patients provided by the collaborating hospital or district health centers, such as the participants’ names, gender, age, location, DAAs prescription, course of treatment, available times for telephone interview, and home telephone number. We also used Excel to record the participants’ responses relating to their barriers to transferring to the hospital for HCV RNA confirmation. The participants’ answers relating to barriers and facilitators regarding hospital referrals were divided into three main categories: (1) accepted referral confirmation, (2) refused referral confirmation, and (3) invalid calls or other factors. These were recorded as percentages by trained nursing staffs (Fig. 1).

## Results

**Fig. 2:**
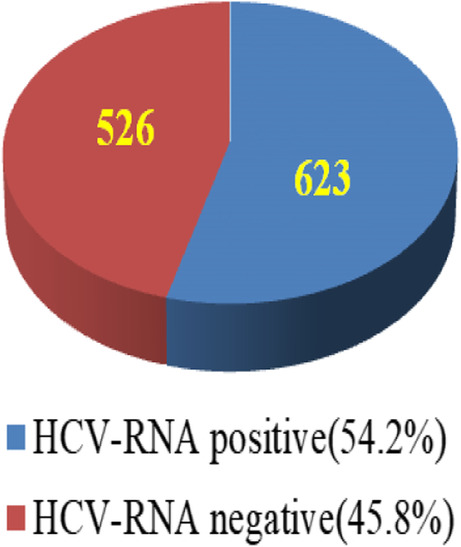
Accept confirmation (*n* = 1149)

**Fig. 3:**
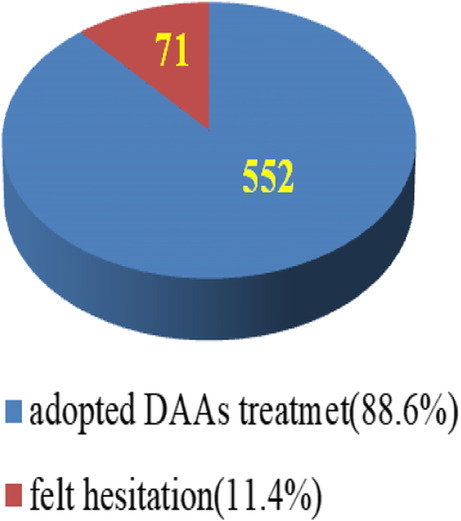
HCV-RNA positive (*n* = 623)

Of the 1795 adults who were serum anti-HCV positive according to the medical records of the collaborating hospital and clinics, 1149 (64%) accepted a transfer to the hospital for HCV confirmation; among the 1149 patients who accepted transfer, 623 (54.2%) were identified as being HCV RNA-positive (Fig. 2). Among them, 552 (88.6%) were willing to undergo DAAs treatment for 2–3 months at the collaborating hospital, while 71 (11.4%) were hesitant to do so (Fig. 3).

The finding shows the facilitators of the participants’ willingness to be transferred in relation to the four main points communicated during the telephone interview: (a) the research team used “treatment is provided for free” to convince participants to accept the hospital referral since some residents had previously undergone treatment with interferon-based injections and experienced a lot of painful side effects because of it. Further, participants were introduced to the free oral medication (DAAs), which had a nearly 100% cure rate and relatively little side effects. A patient qualified for DAAs treatment can save up to 300,000 Taiwanese dollars (TWD) (equivalent to 10,000 USD). Many participants were happy because of this talk. Later, (b) “full understanding of the mechanism of HCV” was explained to the participants. For example, keeping in mind their health-related knowledge and social status, the participants were informed that a “virus like a worm” lives inside their body (liver). However, the virus cannot be easily detected as is it is asymptomatic and is very small; hence, the “worm” needs to be killed or “thrown out of the garden.” During this step, many participants responded that they could understand the mechanism of HCV because of this talk.

Regarding (c) “let us join the national goal and take part in eradicating HCV,” the participants were informed that the government initiated a free program. However, there are limitations as to the number of treatments provided yearly. Thus, the earlier the hepatitis is confirmed, the earlier treatment can be started; otherwise, it will be delayed to the next year. Many participants felt anxious during this step and reported barriers to their receipt of treatment. Regarding (d) “reducing transfer or treatment barriers,” many rural residents mentioned that they faced barriers when transferring to another hospital, such as unfamiliarity with the hospital and transportation problems. The research team assured the participants that there would be someone waiting for them at the gate of the hospital, and that a volunteer (such as a village leader or nursing student) would guide them through each process at the hospital. In addition, the participants were informed that after they began the treatment, the research team would follow up on their response to the drugs. Many participants were reassured by this and immediately said yes.

Table 1 shows the barriers experienced by the 646 participants who did not confirm their treatment; 297 (46%) participants directly refused to be transferred, while 349 (54%) had an invalid telephone number, did not answer, or were dead or hospitalized. Among the 297 patients who refused confirmation, the top four barriers were as follows: (a) the self-perception that they were healthy (*n* = 98; 32.3%) (e.g., “I don’t feel any discomfort at all! There is no need to see doctors! Just save the medical resources for others,” “My liver doesn’t feel painful, and I also need to work every day, so I don’t have spare time to see doctors,” “I don’t think there is a need for me to see doctors; I do not know what hepatitis C is,” “I don’t feel any discomfort on a daily basis; it’s really weird to take medicines for no reason”; (b) mistrust of treatment/healthcare ((*n* = 60; 20.2%) (e.g., “I am used to taking Chinese medicines instead of Western ones; I trust Chinese medicines more than Western ones”); (c) transportation issues (*n* = 52; 17.5%) (e.g., “I did not go to school when I was young; I don’t even know how to take a shuttle bus to the hospital,” and “The hospital is far away from my home … it always takes me at least 2 hours to go see a doctor … without my children’s help, it’s impossible for me to go to the hospital alone”); and (d) work conflict (*n* = 30; 10.1%) (e.g., “I need to work for my whole family; I have 3 work shifts,” “It’s impossible for me to take any time out to see a doctor; just wait and see … ,” “I’m more worried about being jobless than having hepatitis,” and “Without a job, I cannot afford a good life for my family”).

**Table 1:** Barriers to transferring to a hospital for HCV RNA confirmation (*n* = 646)

Variables	N	%
Refusal to be referred for further confirmation (*n* = 297)		
Self-perception that they are healthy	98	32.3
Mistrust of treatment/healthcare	60	20.2
Transportation issues	52	17.5
Work conflict	30	10.1
Worried about the phone scam gang	26	8.8
Previously finished treatment	22	7.4
Difficulty listening	8	2.7
Poor perception of the hospital	1	0.3
Unable to contact (*n* = 349)		
No answer	162	46.4
Invalid telephone number	142	40.7
Death	42	12.1
Hospitalization	3	0.8

## Discussion

Although previous research has indicated that oral DAAs have a high cure rate over a short duration, have few side effects, and are free, many potential HCV patients still face barriers to viral detection and being referred for DAA treatment [5, 6, 17]. Therefore, the present study aimed to understand the implementation facilitators of and barriers to inviting potentially infected patients to accept transfer to a hospital for HCV RNA confirmation and free DAAs treatment in the western coastal Yunlin County [8]. Three key findings emerged from this study. First, it was an efficient strategy to use the anti-HCV data from the collaborating hospital’s medical records for case-finding and to work with the collaborating hospital and clinics. Second, a nurse-led interdisciplinary collaboration involving five elements of referral and a telephone interview with four points for communication significantly facilitated the transfer of potential CHC patients to a hospital and increased their willingness to undergo DAAs treatment. Third, barriers to being referred to a hospital included a high percentage of patients who were unable to be contacted, the patients’ self-perception of being healthy, mistrust of treatment/healthcare, transportation issues, and work conflict.

Recently, in Australia, Pourmarzi et al. [18] demonstrated that a key to achieving the HCV elimination goal is the provision of its treatment in community settings, and that the integration and coordination of care and support provided for both patients and healthcare providers are important processes. The present study lends support to Pourmarzi et al. [18], who reported that successful elements of treatment in community settings include “training and support for healthcare providers, an open referral policy, linkage with or providing outreach services, a person-centered approach, and on-site screening and assessment.” Further, to increase access to treatment for HCV in Australia, White et al. [19] developed a community-based FibroScan and a nurse-led service to assess patients. All patients had treatment prescribed and monitored in primary care, and a telephone follow-up was conducted to confirm that sustained virologic response (SVR) was performed by a clinic nurse. They found that the community-based model facilitates access to HCV treatment in primary care with excellent SVR rates. In Taiwan, the confirmation of HCV and prescription of DAA still requires specialty physicians. The authors of this study strongly recommend that community nurses or primary healthcare providers initiate an integrative program to increase public awareness and promote the referral system for HCV screening and treatment. For instance, there are 368 district public health centers in Taiwan, each with a mastery of the local population’s annual health screening data. If these public institutions could duplicate the present study protocol—wherein once HCV RNA positivity was confirmed by the public health center, the patient would be persuaded to accept free DAAs treatment for 8 ~ 12 weeks depending on his or her genotype—mobilizing such efforts to actively promote DAAs treatment would be an effective integrative program to eliminate HCV in Taiwan.

Unfortunately, many residents with anti-HCV refused to be referred to a hospital for further confirmation because they perceived themselves to be healthy, and appear to have no uncomfortable symptoms. This finding is similar to that of Cheng et al. [20], who conducted a study in remote southern Taiwan. They stated that less than half of confirmed HCV-infected residents received adequate medical care. In addition, Treloar and her colleagues [16] described barriers to HCV care and stigmatization from a social perspective. Previous studies have emphasized the relationship between stigma and adverse health outcomes as well as health access measures. Stigma is a defining factor in HCV treatment, given the association of HCV with the socially demonized practice of injection drug use [16, 21]. Although the present study did not explore the effect of stigma on hospital transfers, it is necessary to comprehensively understand why some people refuse to be referred to a hospital. Nonetheless, when the present researchers approached the residents, some responded that they are worried about the phone scam gang, while others reported having transportation issues or not having enough time because of their need to keep working.

Regarding the barriers of hospital transfer, for rural residents, the hospital is 40–60 km away from the village, and it is particularly difficult for elders to get to without transportation assistance. In addition, it was hard for our research team to reach most patients to notify them because they do not have correct telephone numbers and many residents do not care about DAAs treatment because they feel healthy. Thus, the barriers to being referred to a hospital were clarified. The research team emphasized the communication of the following points to the anti-HCV positive villagers: (1) Since the Taiwanese government fully subsidizes DAAs treatment, patients should seize the opportunity to avail of the free treatment before the annual limit is reached; (2) health is priceless and is the most worthy investment; the sooner you complete your DAAs treatment, the sooner you can rid yourself of the threat of liver cancer; (3) with regard to the 552 patients concerned about adopting the treatment, appropriate health education was given as well as regular reminders to take their medicine; (4) weekly calls were made to remind the patients to return to the hospital for treatment completion; and (5) those who experienced excellent results after undergoing the DAAs treatment were encouraged to promote acceptance of treatment among other patients.

The findings from this study strongly suggest that to achieve the HCV elimination goal of eradicating HCV nationwide, there is a need to make vigorous efforts to enhance screening coverage, educate people about new information relating to liver disease, reduce the barrier of work conflict, and use the media to promote related policy and hospital referrals. The present findings showed a high percentage of invalid or disconnected home telephone numbers (*n* = 304). This may be due to wrong numbers being included in the medical records provided by the collaborating hospital and clinics. This could also be attributed to the fact that the patients’ health screenings were conducted many years ago, and they changed their telephone numbers in recent years. Therefore, to increase case findings, it is crucial to collect correct information through multiple sources (e.g., ask for help from the district government, or visit village leaders and make home visits).

This study has some limitations that should be noted. First, although the research team encouraged all healthcare providers in the outpatient clinics to survey medical records and refer each anti-HCV patient to the collaborating hospital for HCV confirmation, some medical personnel from the private sector had reservations regarding this approach (e.g., some of them felt that it was not so important). In addition, there were no incentives for primary healthcare providers from public health centers to make transfer referrals. Consequently, this may limit the case findings from rural areas. Second, the high percentage of invalid telephone numbers and failure to answer the telephone limits the referrals and the effectivity of the approach used in the study. Thus, it is necessary to reestablish valid telephone connections via the public and private sectors. Third, Taiwan has launched the NHI system and covers almost all of the Taiwanese population. However, since each item for the antibody test costs less than 7 USD, while each HCV RNA confirmation costs over 70 USD, the government uses two steps to confirm an active HCV infection. This procedure increases the barriers to hospital transfer for HCV RNA confirmation and DAAs treatment of CHC patients. Therefore, we strongly suggest that confirmatory testing (HCV RNA) should be done locally, with referrals for those that are HCV RNA positive.

## Conclusion

The findings of this study show that the interdisciplinary collaboration approach, with five elements of referral and a telephone interview wherein four points for communication are emphasized, significantly influenced the patients’ acceptance of hospital referrals and their willingness to undergo a new drug treatment in endemic rural areas of Taiwan. In addition, it was an efficient strategy to use medical records provided by the collaborating hospital, clinics, and district health centers to identify residents who were anti-HCV positive and increase case findings. Future research should focus on reducing barriers to hospital transfer and treatment in disadvantaged areas, such as transportation issues, work shifts, invalid telephone numbers, and incorrect self-perceptions of being healthy.

## Data Availability

The datasets analyzed during the current study are not yet publicly available but are available from the corresponding author on reasonable request.

## Abbreviations

HCV: Hepatitis C virus
CHC: Chronic hepatitis C
anti-HCV: Hepatitis C virus antibody positive
DAA: Direct-acting antiviral agents
